# Advancing the diagnosis of rare neuromuscular and neurological diseases through the collaborative Solve-RD research framework

**DOI:** 10.1177/22143602261460689

**Published:** 2026-07-15

**Authors:** Lisa-Sophie Wüstner, Kornelia Ellwanger, Nika Schuermans, German Demidov, Kiran Polavarapu, Leslie Matalonga, Steven Laurie, Solve-RD DITF-RND, Solve-RD DITF-EURO-NMD, Ana Töpf, Katja Lohmann, Holm Graessner

**Affiliations:** 1 Institute of Medical Genetics and Applied Genomics, 9188 University of Tübingen, Tübingen, Germany; 2 Center for Medical Genetics, 60200 Ghent University Hospital, Ghent, Belgium; 3 Children’s Hospital of Eastern Ontario Research Institute, Ottawa, Canada; 4 560773 Centro Nacional de Análisis Genómico (CNAG), Barcelona, Spain; 5 John Walton Muscular Dystrophy Research Centre, Translational and Clinical Research Institute, Newcastle University and Newcastle Hospitals NHS Foundation Trust, Newcastle Upon Tyne, UK; 6 Institute of Neurogenetics, 9191 University of Lübeck, Lübeck, Germany; 7 Centre for Rare Diseases, 9188 University of Tübingen, Tübingen, Germany

**Keywords:** Solve-RD, rare neuromuscular diseases, rare neurological diseases, multi-omics, long-read sequencing, optical genome mapping, RNAseq, diagnostic reanalysis

## Abstract

Rare neuromuscular and neurological diseases (NMDs and RNDs) present diagnostic challenges due to their clinical heterogeneity and genetic complexity. Despite the advancements in next-generation sequencing (NGS) and other high-throughput genomic technologies, a significant proportion of patients with NMDs and RNDs remain undiagnosed. This is primarily due to genetic heterogeneity, the presence of novel or private variants, and incomplete variant detection by short-read sequencing platforms. The Solve-RD project, a pan-European initiative funded by the Horizon 2020 programme, established a robust interdisciplinary framework integrating expert clinical and bioinformatics teams through Data Interpretation Task Forces (DITFs) and Data Analysis Task Force (DATF). Focusing on previously undiagnosed NMD and RND patients, Solve-RD implemented a systematic reanalysis of exome/genome data. For specific cohorts, various omics approaches were added, including long-read genome sequencing, RNA sequencing, and optical genome mapping. This collaborative framework significantly improved diagnostic yield in RND and NMD cohorts and led to the identification of novel pathogenic variants and mechanisms. The Solve-RD model exemplifies how structured expert collaboration, data sharing and harmonisation, and cutting-edge multi-omics technologies can overcome current diagnostic limitations in rare disease research.

## Introduction

Rare neuromuscular (NMD) and neurological diseases (RND) represent a broad and heterogeneous group of inherited disorders affecting millions of individuals worldwide.^1–3^ While each disorder is individually rare, collectively they contribute to substantial morbidity, often with delayed diagnoses, and limited access to targeted therapies.^4^ Conventional diagnostic approaches, including whole exome sequencing (WES), often fail to identify a causal variant, leaving over half of patients without a definitive diagnosis.^5,6^ Contributing factors include the extensive clinical and genetic heterogeneity, the presence of novel or private variants, and the incomplete detection of certain pathogenic variants, such as repeat expansions, complex structural variants by short-read sequencing technologies, as well as the interpretation of deep intronic variants.^7,8^

To address these challenges, the Solve-RD (Solving the Unsolved Rare Diseases) project, funded by the European Union under Horizon 2020, established a pan-European collaborative framework.^9^ Its core innovation is the integration of expert clinical groups, i.e., advanced genomic analysts of the Data Analysis Task Force (DATF) with the Data Interpretation Task Forces (DITFs). The DITFs incorporate the clinical expertise from European Reference Networks (ERNs). These are networks of clinical healthcare providers established in 2017 by the European Union, working together to diagnose and treat rare or complex disease patients which require highly specialised knowledge and resources.^10^ Notably, Solve-RD was the first collaborative research project to which several partners from various ERNs contributed.^11^

Together, the DATF and the DITFs reanalyzed existing genomic datasets, including WES and short-read whole-genome sequencing (SR-WGS) data, and performed primary analyses and integrative interpretation of newly generated multi-omics data for previously unsolved cases. The Solve-RD analysis effort thus comprised two complementary approaches applied to previously unsolved cases: reprocessing of existing genomic data through updated pipelines, and the generation of novel multi-omics data from available biosamples, both incl. Interpretation of the resulting variants. The novel omics approaches included the generation of short- and long-read whole-genome sequencing (SR-/LR-WGS), optical genome mapping (OGM), transcriptomic analyses such as short- and long-read RNA sequencing (SR-/LR-RNAseq), and epigenomic profiling. Key components of the workflow encompassed multidisciplinary team discussions (MDTs) and intensive cross-specialty workshops, known as Solvathons, designed to foster structured collaboration and problem-solving.^12,13^ This cooperative model has increased diagnostic yield, accelerated the resolution of previously unsolved cases, and provided a scalable blueprint for future initiatives. Within the Solve-RD project, the systematic reanalysis strategy led to new diagnoses in 506 families - accounting for 8.4 % of the pan-European cohort, which included disease groups covered by four ERNs i.e. NMD, RND but also ITHACA for rare neurodevelopmental disorders and GENTURIS for rare tumor syndromes.^12^ Importantly, the impact of Solve-RD reaches beyond the end of its funding period, enabling additional diagnoses in RND and NMD cases. Building on the Solve-RD publication by Laurie et al.,^12^ which reported diagnostic outcomes across all four ERN disease cohorts, this manuscript focuses specifically on the RND and NMD subcohorts, revisiting and expanding the findings reported for this group. Here, we project specific subcohorts within the Solve-RD framework and analyze the proportion of solved cases based on the current diagnostic status recorded in the Phenostore instance of the RD-Connect Genome-Phenome Analysis Platform (GPAP), the project’s centralized system for capturing clinical information and diagnostic outcomes.^14^ We detail the implementation of the Solve-RD framework and its impact during the project phase, and present additional diagnoses achieved through continued iterative reanalysis, novel-omics applications, and new disease-gene discoveries made since the initial publication, providing an updated and more granular account of the ongoing contribution of the Solve-RD framework to solving previously undiagnosed NMD and RND cases.

## Main

### Establishing clinical cohorts and expertise

The European Reference Networks for Rare Neurological Diseases (ERN-RND)^2^ and for Neuromuscular Diseases (ERN EURO-NMD) provided essential clinical expertise and well-characterised patient cohorts for the reanalysis and novel-omics approaches in Solve-RD.^9,11^ Through their transnational infrastructure, both ERNs enabled the systematic recruitment of patients with unsolved RNDs and NMDs, harmonised phenotypic and genetic data collation for reanalysis, and facilitated the generation of novel-omics datasets from available biosamples. Corresponding to each disease group, a dedicated DITF has been established, which will be further described in the Boxes 1 and 2 for RND and NMD, respectively. Full lists of members and their affiliations appear in the Supplementary Information.

**Box 1. DITF-RND**

The DITF-RND network comprises 71 clinical and genetic experts in the field of neurogenetics, affiliated with 16 genetic diagnostic and/or research centers distributed across Belgium, France, Germany, Greece, Hungary, the Netherlands, Slovenia, Spain, Turkey, and the United Kingdom. The areas of expertise of the DITF members span multiple aspects of neurogenetics, including hereditary ataxias and spastic paraplegias, paroxysmal neurological disorders, movement disorders, leukodystrophies, peripheral neuropathies, neurodegenerative diseases, and pediatric neurology. The main focus of the reanalysis and novel-omics studies was on ataxia and spastic paraplegia, where multi-omics strategies, such as long-read sequencing and RNAseq, are crucial in uncovering hidden genetic variants, such as repeat expansions and deep intronic disease-related variants, that were not detected in the initial genomic analysis.

**Box 2. DITF-NMD**

The DITF-NMD network unites 73 clinical genetics experts, affiliated with 19 centers in Belgium, Canada, Finland, France, Germany, Italy, Portugal, Spain, and the United Kingdom. Overall, the most frequently used Human Phenotype Ontology (HPO) terms to describe affected individuals within the NMD cohort, related to muscle weakness, myopathy, and abnormal muscle morphology. The clinical composition of the novel-omics studies conducted by the DITF-NMD network focused on a wide range of distinct and complex disorders, such as Ehlers-Danlos syndrome, Oculopharyngodistal myopathy (OPDM), contractural phenotypes, dominantly inherited rhabdomyolysis, myopathies (including distal, axial, and early onset vacuolar subtypes), proprioceptive disorder, neuropathy and mitochondrial diseases.

**Figure 1.**
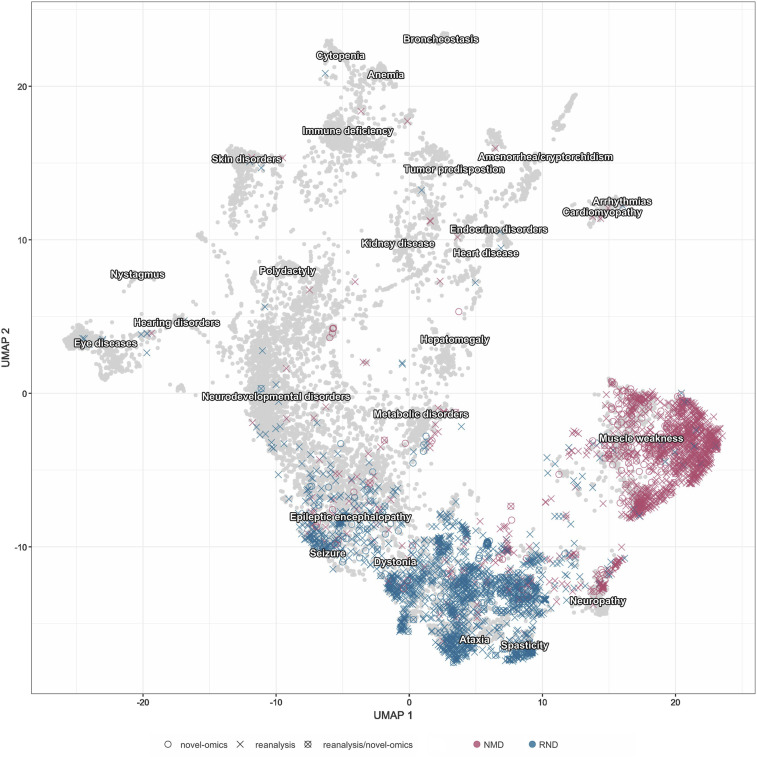
Uniform manifold approximation and projection visualisation of patient phenotypes based on HPO term similarities. This figure demonstrates the phenotypic diversity and clustering patterns within the Solve-RD RND and NMD cohorts. Gray background dots represent all Online Mendelian Inheritance in Man (OMIM) diseases, providing a comprehensive disease phenotype reference for comparison. Color coding indicates patient assignment to RND or NMD cohorts, while different symbols indicate the investigative strategy applied in Solve-RD. The distribution of patients across phenotypic space illustrates the substantial clinical and genetic heterogeneity characteristic of rare neurological and neuromuscular diseases, underscoring the need for tailored diagnostic approaches. Due to the high density of overlapping points, symbol distinction may be limited; this figure should be interpreted as an illustration of phenotypic diversity rather than a basis for inferring diagnostic patterns. Figure created with https://github.com/GermanDemidov/phenotypes_plot.

We used visualisation of phenotypic clustering based on the HPOs of the included patients to present the phenotypes distribution (Figure 1). The Uniform Manifold Approximation and Projection Visualisation (UMAP) analysis demonstrates that RND and NMD patients predominantly cluster apart, with minor overlap observed between the two groups. The choice of methodological approach (reanalysis, novel-omics, or their combination) for each patient in the Solve-RD cohort was determined by both (i) association of the ability of methodological approaches to detect specific variant types and a-priori disease group knowledge (e.g. for unsolved ataxia cases, long read sequencing was done based on the previous knowledge that repeat expansions play an important role in genetic disease causation^15^ and (ii) by the availability of suitable biosamples or existing data. Therefore, and because specific phenotypic features only to a limited extent determined the choice of methodological approach, patient cohorts analyzed with different strategies do not form separate clusters within the UMAP visualisation. This explains why there is no clear segregation of the approaches within the two cohorts, reflecting a pragmatic selection process driven by practical resource considerations.

### The Solve-RD analysis and interpretation workflow for NMD and RND patients

**Figure 2.**
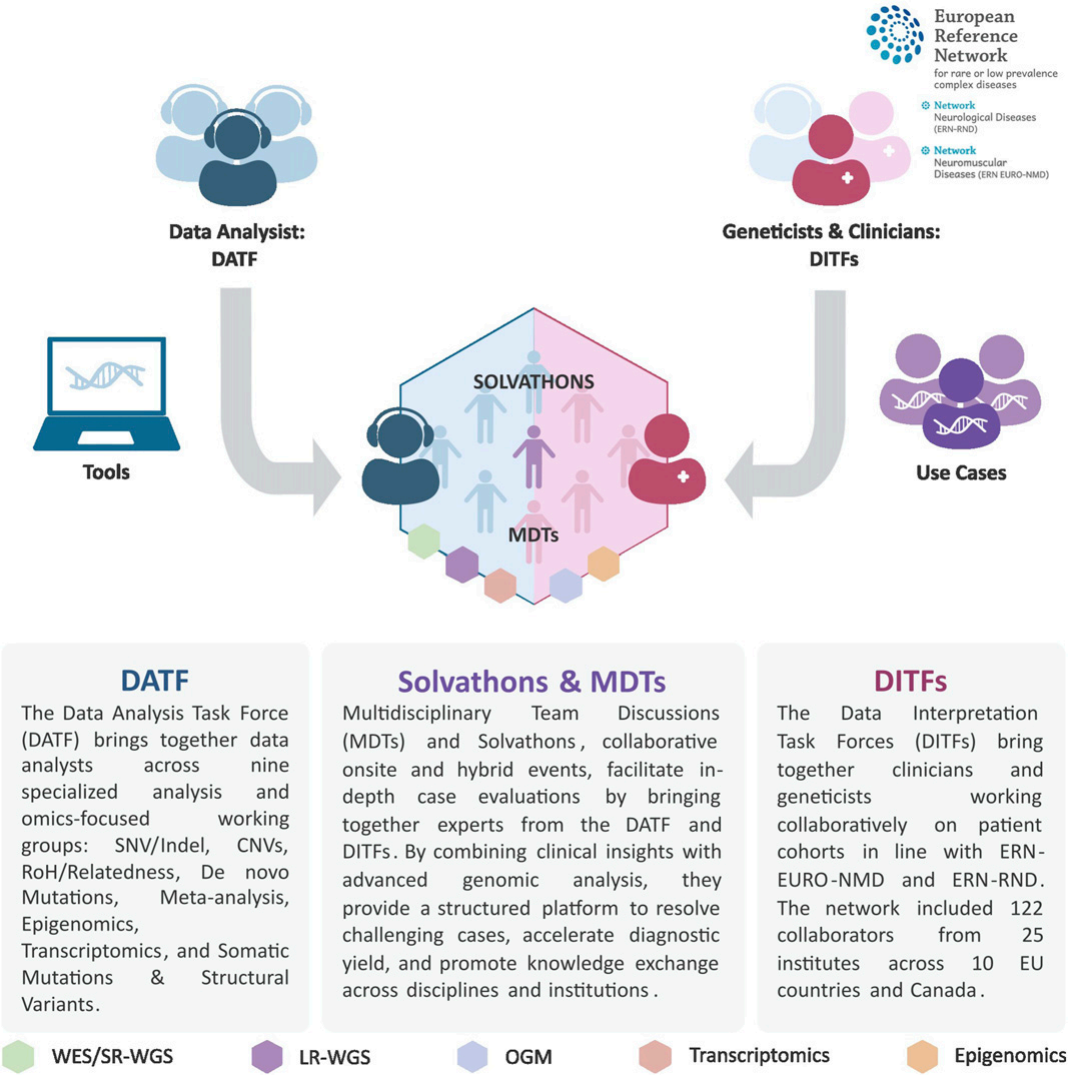
Solve-RD DATF-DITF workflow. This figure illustrates the systematic integration of computational and clinical expertise central to the Solve-RD diagnostic framework. The schematic depicts how data analysts from the DATF collaborate with geneticists and clinicians within the DITFs through structured multidisciplinary events (Solvathons & MDTs) to address complex genetic cases. Computational reprocessing is performed by the DATF, applying updated bioinformatic pipelines and variant callers to existing raw sequencing data (WES, SR-WGS) to identify variants previously missed due to technical limitations. Variant evaluation is conducted jointly across both teams. Where standard reanalysis approaches remain inconclusive, novel multi-omics data types, including LR-WGS, OGM, and RNA sequencing, are generated and integrated to provide functional and structural evidence resolving ambiguous cases. This coordinated approach leverages complementary expertise and iterative analysis across multiple genomic strategies to overcome diagnostic bottlenecks and enable comprehensive variant detection and interpretation in rare neurological and neuromuscular diseases. Schematic is adapted from Zurek et al.^9^ *Abbreviations: WES: Whole Exome Sequencing; SR-WGS: Short-Read Whole Genome Sequencing; LR-WGS: Long-Read Whole-Genome Sequencing; OGM: Optical Genome Mapping.*

The Solve-RD systematic (re)analysis and interpretation pipeline builds on a coordinated workflow including two major functional groups: the Data Analysis Task Force (DATF) and the Data Interpretation Task Forces (DITFs) (Figure 2). The DATF consists of bioinformaticians and molecular geneticists who apply standardized yet most innovative (gen)omic analysis pipelines and, in parallel, develop and integrate novel approaches to overcome the inherent limitations of standard workflows and tools. The research work of the DATF is supported by a centralised and interconnected data infrastructure (RD-Connect Genome-Phenome Analysis Platform (GPAP),^14^ MOLGENIS Rare Disease Data about Data (RD3)^16^ and the European Genome-Phenome Archive (EGA)^17^) that ensures data harmonisation and secure access. DITFs, composed of disease-specific clinical experts, interpret these results in light of detailed phenotypic and pedigree information. In addition to the systematic DATF-DITF workflow, many contributing partners perform local or GPAP-based analyses on specific families of interest. These *ad hoc* expert reviews further contributed to novel diagnoses and gene discoveries.

Central to the Solve-RD (re)analysis approach are MDTs and Solvathons, where experts from both task forces convene, onsite or virtually, for in-depth case evaluation and integrative diagnostics.^13^ These collaborative workshops enabled real-time discussion between clinicians and analysts and, furthermore, function as training and knowledge-sharing vehicles.

### Systematic reanalysis of RND and NMD patients results in new diagnoses

In the Solve-RD project a massive data collection for reanalysis of existing data was undertaken, involving clinical and genomic datasets of a total of >23,000 individuals from >13,000 rare disease families across Europe. The systematic reanalysis of an initial subset of 6,004 families facilitated the diagnosis of more than 500 patients.^12^ As part of this effort, genomic data from 4,138 affected individuals, representing 3,788 RND and NMD families, were reanalyzed. This subset accounted for over 60 % of all affected individuals and families in the respective subcohort, with the remaining families reflecting disease groups covered by ERN-ITHACA and ERN GENTURIS. All available WES and SR-WGS datasets were harmonised and reprocessed using updated alignment, variant calling and annotation tools, and prioritisation strategies.

Of the diagnoses reported by Laurie et al.^12^ for systematic reanalysis across all Solve-RD disease groups, the RND and NMD subcohort accounted for 441 newly diagnosed families. These comprised 303 RND and 138 NMD families identified through systematic reanalysis. Further 103 RND and NMD families were diagnosed through local ad hoc expert review conducted in parallel to and independently of the standardised systematic reanalysis, which resulted in an overall diagnostic yield of 11.6 % for this subcohort (Table 1). For RND families specifically, the yield increased to 13.4 %, while NMD families reached 9.1 %. Within the systematic reanalysis of existing genomic data, families were reassessed for a comprehensive range of variant types, including single nucleotide variants and insertions/deletions (SNV/InDels), mitochondrial DNA (mtDNA) variants, copy number variants (CNVs), structural variants (SVs), mobile element insertions (MEIs), and short tandem repeat expansions (STRs).^12^ Notably, mtDNA and STR reanalysis proved particularly impactful for RND and NMD families, with over 81 % of all mitochondial variants reported as causative by Laurie et al. originating from RND and NMD cases.^12^ The diagnostic value of dedicated mtDNA reanalysis pipelines, particularly in neuromuscular and neurological disease cohorts was also highlighted in Ratnaike et al. 2025.^18^ Similarly, all families who received a diagnosis via STR analysis belonged exclusively to the RND or NMD entities.^12^ In many cases, pathogenic variants had previously been missed due to limitations of earlier pipelines or due to their interpretation as VUS, incomplete and/or not updated databases or for being in genes not associated with disease at the time of analysis.^12,18–23^ Crucially, the reassessment of previously identified variants was informed by insights gained from newly solved cases, enabling a molecular diagnosis. Furthermore, 160 families received a candidate diagnosis, highlighting the extended potential clinical impact of the systematic reanalysis.

**Table 1.** Overview of RND & NMD families solved by reanalysis during Solve-RD^12^ and beyond. The total numbers of families are indicated in bold.

​		Total	RND	NMD
Families enrolled in systematic reanalysis approach^12^	​	**3788**	2271	1517
Families reported as solved in Laurie et al.^12^	total	**441**	303	138
	Ad Hoc Expert Reviews	**103**	61	42
	Systematic Reanalysis	**338**	242	96
Additional Solved Families (Post Solve-RD)	​	**134**	82	52
**Total solved families**	​	**575**	**385**	**181**

While this reflects the status reported by Laurie et al.,^12^ which is based on the data collected, reanalyzed and interpreted by early 2023, the joint effort to solve cases was continued. Over the subsequent three years, additional 134 cases were solved (Table 1, ‘Post-Solve-RD’). Overall, among 3,788 previously undiagnosed families submitted for reanalysis, 575 have received a molecular diagnosis, increasing the diagnostic yield for RND and NMD cases to 15.2 %. The increasing practice of re-evaluating previously unsolved cases across European clinical centers highlights both the ongoing value and the evolving role of genetic reanalysis, alongside the application of novel technologies in diagnostics. This shift, driven by approaches like novel-gene identifications, reverse pheno-genotype correlations, systematic reclassification of variants and innovative omics techniques, demonstrates a growing recognition that initial negative results should be seen as provisional, reinforcing the need for continuous refinement of diagnostic strategies.

A notable example among the newly solved cases was the discovery of pathogenic GAA repeat expansions in the Fibroblast Growth Factor 14 (*FGF14*) gene as a causative mechanism for late-onset ataxia.^24,25^ These *FGF14* repeat expansions, identified by specific screenings as described in the referenced publications, accounted for approximately 15 % (21 out of 134) of the solved ‘Post Solve-RD’ families. and exemplify the benefit of the Solve-RD cohort strategy: namely that a newly detected genetic defect can be screened in an established cohort of unsolved ataxia cases.

### Multi-omics integration

**Figure 3.**
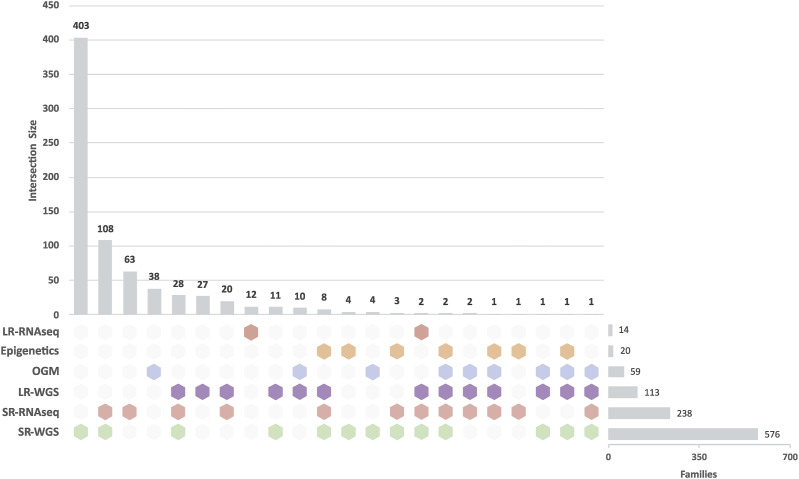
Multi-omics integrative analysis of RND and NMD families. This UpSet plot demonstrates the breadth and complexity of (gen)omic approaches applied across the Solve-RD cohorts. The visualization illustrates the distribution of families stratified by specific combinations of sequencing and novel-omics-based analysis techniques. Coloured hexagons indicate whether a given technique was applied. Horizontal bars indicate the number of families receiving a specific combination of methods. Vertical bars indicate the total number of families analyzed with each technique. The multiple overlapping combinations highlight the tailored, case-by-case approach adopted by Solve-RD to address the diagnostic heterogeneity of rare neurological and neuromuscular diseases. *Abbreviations: LR-RNAseq: Long-Read RNA Sequencing; SR-RNAseq: Short-Read RNA Sequencing; OGM: Optical Genome Mapping; LR-WGS: Long-Read Whole-Genome Sequencing; SR-WGS: Short-Read Whole-Genome Sequencing.*

For selected cohorts, among those prioritising structural variant analysis, Solve-RD implemented an expanded multi-omics strategy that included the generation of SR-/LR-WGS (HiFi-PacBio), OGM, RNAseq, as well as additional approaches such as epigenetic profiling, alone or in combination (Figure 3). In total, 2,347 biosamples from 1,707 participants across 751 RND and NMD families were included in this integrative novel-omics analysis approach (Table 2). While reanalysis of existing genomic data was reported in the Solve-RD flagship publication by Laurie et al.,^12^ the integrative multi-omics findings presented here represent a distinct and complementary body of work carried out during the project. Some of these analyses, particularly epigenetic profiling and the integration of multi-omics data, started later in the project and are still ongoing long after its formal conclusion. This continued work highlights both the complexity of these datasets and the potential of the resource generated for uncovering previously unresolved diagnoses. These data have either been published in subsequent manuscripts or remain unpublished at the time of writing. Collectively, they provide a comprehensive and evolving assessment of diagnostic outcomes for RND and NMD families investigated through multi-omics approaches within the Solve-RD framework.

**Table 2.** Overview of biosamples, participants and families that received novel-omics analysis in Solve-RD.

Disease group	Total number of biosamples	Total number of individuals assigned to novel-omics	Total number of families assigned to novel-omics
NMD	1067	911	362
RND	1280	796	389
**Total**	**2347**	**1707**	**751**

Trio LR-WGS analysis was performed in a subcohort of families without a genetic diagnosis despite extensive prior testing, as previously described.^26^ Of the 75 RND and NMD families included in this cohort, 11 families (14.7 %) have received a confirmed diagnosis, while 4 additional families (5,3%) were found to have strong candidate variants.^26^ Long-read technology revealed rare inherited and *de-novo* variants, including deep intronic variants, structural rearrangements, and repeat expansions missed by previous analyses. Transcriptome profiling through RNAseq analysis was applied to perform expression and splicing analysis of both known and novel disease genes, and included cases where candidate variants were validated and cases with no prior candidate variant.^27,28^ In several of these cases, the RNAseq data clarified VUS interpretation and allowed variant reclassification by demonstrating aberrant expression, aberrant splicing, or allele-specific expression. Overall, across ERNs this effort resulted in molecular diagnoses for 19 out of 248 families (7.7 %) for which DNA analyses (WES/WGS reanalysis or novel-SR-WGS analysis) had been inconclusive.^29^ RNDs and NMDs comprised a substantial proportion of these cohorts, and pathogenic variants were identified in 16 out of 151 RND/NMD families (10.6 %). Noteworthy, transcriptomic analysis in RND and NMD yielded an even higher diagnostic rate than in the cross-ERN cohort. Across all RND/NMD families analysed by novel-omics approaches within the Solve-RD framework (Figure 3), the proportion of solved cases was assessed based on the current diagnostic status recorded in the GPAP Phenostore instance. Families investigated using SR-WGS alone showed a solved rate of approximately 16 % (64 out of 403), whereas inclusion of SR-RNAseq was associated with higher yields of around 26 % (28 out of 108). The highest solved rate, exceeding 40%, was observed in the subset of families analyzed using an integrated strategy combining SR-WGS, LR-WGS, and SR-RNAseq (12 out of 28). These observations suggest that tailored, integrated multi-omics strategies can enhance diagnostic outcomes in carefully selected cases, particularly those with complex presentations or inconclusive standard testing. However, conclusions must be drawn cautiously, as the aggregated case numbers do not allow direct attribution of individual diagnoses to a specific omics modality or combination. Nevertheless, this trend is consistent with prior findings, including the study by Estévez-Arias et al., which demonstrated that integrating genome and transcriptome sequencing improves diagnostic resolution in exome-negative childhood-onset neuromuscular diseases, particularly by revealing pathogenic splice-altering and regulatory variants missed by DNA-based analyses alone.^27^

### Solvathons

Solvathons are formalised, novel-omics focussed workshops that operationalise the DATF-DITF collaborative framework for solving the unsolved. Across all four Solvathon events organised in Solve-RD, a total of 1,069 unique families were included for joint analysis.^13^ Of these, 601 families were part of the RND and NMD cohorts, representing a substantial proportion of the overall dataset. The coordinated efforts of the Solvathon workflow led to the resolution of 28 participant cases, with 12 of these solved cases from the RND and NMD cohorts. A particularly illustrative example is the discovery of a pathogenic repeat expansion in *NOP56* in a family with a previously undiagnosed cerebellar ataxia. Using RNAseq data during a Solvathon, decreased *NOP56* expression was detected, which prompted targeted follow-up analyses that confirmed the repeat expansion, as reported by Yépez et al.^13^ This case highlights how Solvathons can enable the detection of complex variants, such as repeat expansions, that are often missed by standard analyses and underscores the value of collaborative, multi-omics review, particularly for RND cases.

## Discussion

The diagnostic advances achieved through Solve-RD underscore the power of its interdisciplinary framework and data-sharing infrastructure. The DITF-DATF collaboration framework not only enabled comprehensive reanalysis and interpretation of rare disease cases by integrating bioinformatics, clinical expertise, and emerging genomic evidence, but also improved knowledge sharing and communication.

Nevertheless, significant gaps in the genetic diagnosis of rare neurological and neuromuscular diseases still remain. As sequencing technologies evolve, the increase in diagnostic yield should be viewed not as a static achievement, but as part of an ongoing process. Also, with new advancements in genomic analysis and interpretation tools and methodologies, there will be a need for continued reanalysis of existing datasets. Implementing this in routine practice will require consensus on reanalysis criteria, adequate resourcing, and close laboratory-clinical communication, alongside further development of automated pipelines and AI-driven variant prioritisation and phenotype-genotype matching, tools that could make systematic reanalysis feasible even in resource-limited settings, provided they undergo rigorous validation.

The success of novel-omics approaches, beyond standard clinical exomes, highlights their growing importance in the diagnostic process, particularly with respect to structural and deep intronic variants that were previously missed. Growing genetic knowledge, facilitated by tight DITF-DATF collaborations, led to new genetic diagnoses. These MDT collaborations continue beyond the Solve-RD project. For instance, a combination of novel-omics, *i.e.* OGM and transcriptomics, provided a diagnosis for a patient with a complex movement disorder comprising dystonia, ataxia, tremor, and spasticity. The disease-causing mutation was found to be a two-exon deletion in *KIF1C* that had not been called by analysing WES data (Thomsen et al., under review). Another example for the added value of long-read analyses is provided by Trinh and colleagues in 2024, who showed that phasing in recessive disease-linked genes, such as *PRKN*, where seemingly adjacent exon deletions turned out to be on different alleles, can lead to disease-explaining compound-heterozygous pathogenic variants.^30^

Furthermore, while LR-WGS has provided insights into previously elusive pathogenic variants, the integration of transcriptomic data through RNAseq has further advanced our ability to interpret VUSs. This combination of DNA and RNA data provides a more holistic view of the genetic landscape, offering more robust evidence for variant pathogenicity. Multi-omics strategies are no longer confined to research settings: as costs decrease and workflows become more standardised, their integration into routine clinical diagnostics is increasingly feasible. Nevertheless, scalability remains a challenge, requiring investment in bioinformatics infrastructure, trained personnel, and robust data-sharing frameworks, areas in which Solve-RD has done important groundwork.

A critical aspect of the Solve-RD approach is the continued collaboration between clinicians and geneticists within the DITF-DATF framework, especially through the Solvathons and MDTs. The interdisciplinary Solvathon workshops not only facilitate real-time diagnostics but also promote knowledge-sharing and capacity building, ensuring that both clinical and genomic expertise evolve together. This model of structured, multidisciplinary collaboration offers a template for how multi-omics diagnostics could be sustainably implemented in clinical practice more broadly, particularly for healthcare systems seeking to scale rare disease diagnostic programmes without duplicating efforts.

Beyond immediate diagnostic gains, the Solve-RD data resource represents a powerful and sustainable foundation for future rare disease research, facilitating both novel disease-gene discoveries and cross-cohort analyses, such as the identification of *CD99L2* as a cause of X-linked spastic ataxia^31^ and the comprehensive assessment of *TTN* variants in unsolved neuromuscular disease cohorts.^32^

The Solve-RD data is stored at the EGA and can be made available to other rare disease researchers via a controlled access mechanism (https://solve-rd.eu/results/solve-rd-data/). The comprehensive data and infrastructure resource facilitates further and future research.^16^

## Conclusion

The Solve-RD project has set a new benchmark for collaborative diagnostics in rare diseases, demonstrating that with the right combination of genomic technologies and clinical expertise, many previously undiagnosed patients can receive a definitive genetic diagnosis. This approach has paved the way for a better understanding of rare neurological and neuromuscular diseases, some of which were not recognised until the application of multi-omics strategies. The findings underscore the importance of continued innovation in diagnostic technologies and the value of data reanalysis as part of a dynamic diagnostic process.

The broader implication of Solve-RD’s success is its potential scalability across other rare disease networks globally. The integrated model employed by Solve-RD offers a framework that can be adapted to other disease areas, fostering a more efficient and comprehensive approach to rare disease diagnostics. As the scientific community continues to refine both the tools for genetic analysis and the methods for interpreting complex data, the integration of novel-omics approaches will play an increasingly important role in the accurate diagnosis and understanding of rare diseases.

Lastly, it is essential to recognise that the value of such collaborative projects extends beyond the immediate diagnostic outcomes. The data generated, new collaborations established and the lessons learned provide resources for future research, including the development of more personalised and effective therapies for rare disease patients.

## Supplemental material

Supplemental material - Advancing the diagnosis of rare neuromuscular and neurological diseases through the collaborative Solve-RD research framework Supplemental material for Advancing the diagnosis of rare neuromuscular and neurological diseases through the collaborative Solve-RD research framework by Lisa-Sophie Wüstner, Kornelia Ellwanger, Nika Schuermans, German Demidov, Kiran Polavarapu, Leslie Matalonga, Steven Laurie, Solve-RD DITF-RND, Solve-RD DITF-EURO-NMD, Ana Töpf, Katja Lohmann, and Holm Graessner in Journal of Neuromuscular Diseases.

Supplemental material - Advancing the diagnosis of rare neuromuscular and neurological diseases through the collaborative Solve-RD research framework Supplemental material for Advancing the diagnosis of rare neuromuscular and neurological diseases through the collaborative Solve-RD research framework by Lisa-Sophie Wüstner, Kornelia Ellwanger, Nika Schuermans, German Demidov, Kiran Polavarapu, Leslie Matalonga, Steven Laurie, Solve-RD DITF-RND, Solve-RD DITF-EURO-NMD, Ana Töpf, Katja Lohmann, and Holm Graessner in Journal of Neuromuscular Diseases.

## Data Availability

Solve-RD data is available at the European Genome-Phenome Archive (https://ega-archive.org/, under the Solve-RD study EGAS00001003851), following approval from the Solve-RD Data Access Committee (https://solve-rd.eu/results/solve-rd-data/).
