# Supplementary material for: Advancing the diagnosis of rare neuromuscular and neurological diseases through the collaborative Solve-RD research framework
Source: J Neuromuscul Dis. 2026 Jul 15:22143602261460689. Online ahead of print. doi: 10.1177/22143602261460689 (PMC13438582; doi:10.1177/22143602261460689)
Supplement: Supplementary file 1 [file sj-pdf-1-jnd-10.1177_22143602261460689.pdf]

Solve-RD DITF-RND

Jonathan Baets[1-3], Peter Balicza[4], Selina Banu[5], Ayşe Nazlı Başak[6], Danique Beijer[7], Frederik Braun[8], Elisa Cali[5], Ana Laura Cazurro[9], Viorica Chelban[5], Patrick Chinnery[10, 11], Giulia Coarelli[12], Jonathan de Winter[1-3], Bart Dermaut[13-15], Minas Drakos[16], Alexandra Dürr[17-21], Stephanie Efthymiou[5], Lisa-Marie Erlandsson[22, 23], Fei Gao[10], Holm Graessner[24, 25], Lena Guillot-Noel[12], Tobias Haack[24], Michael G. Hanna[5], Dimitri Hemelsoet[14, 26], Holger Hengel[22, 23], Rebecca Herzog[27, 28], Peter Heutink[22, 23], Rita Horvath[10], Henry Houlden[5], Erik-Jan Kamsteeg[29], Josua Kegele[30], Melanie Kellner[22, 23], Christoph Kessler[22, 23], Evgenia Kokosali[16], Ingrid Kolen[31], Holger Lerche[30], Katja Lohmann[32], Alfons Macaya[9, 33], Francesca Magrinelli[34], Anna Marce Grau[9], Ales Maver[35], Judit Molnar[4], Heba Morsy[5, 36], Alexander Münchau[27], Juan Dario Ortigoza-Escobar[37-39], Martje G. Pauly[27, 28, 32], Borut Peterlin[35], Martin Regensburger[40], Selina Reich[7, 23], Olaf Riess[24, 25], Jonathan Rohrer[41], Vincenzo Salpietro[5], Ulrike Schara-Schmidt[8], Ludger Schöls[22, 23], Nika Schuermans[13-15], Rebecca Schüle[22, 31], Giovanni Stevanin[17-19, 21, 42], Tipu Sultan[5], Matthis Synofzik[7, 23], Huma Tariq[5], Vincent Timmerman[2, 43], Liedewei van de Vondel[1, 2, 43], Bart van de Warrenburg[44, 45], Nienke van Os[44, 45], Jana Vandrovcova[5], Hannah Verdin[13], Edgard Verdura[9], Sarah Wiethoff[5], Carlo Wilke[7, 23], Jürgen Winkler[40], Jishu Xu[22-24], Ioannis Zaganas[16].

Affiliations

1. Translational Neurosciences, Faculty of Medicine and Health Sciences, University of Antwerp, Belgium.
2. Laboratory of Neuromuscular Pathology, Institute Born-Bunge, University of Antwerp, Antwerpen, Belgium.
3. Neuromuscular Reference Centre, Department of Neurology, Antwerp University Hospital, Antwerpen, Belgium.
4. Institute of Genomic Medicine and Rare Diseases, Semmelweis University, Budapest, Hungary.
5. Department of Neuromuscular Diseases, UCL Queen Square Institute of Neurology, London, UK.
6. Koç Universıty,School of Medicine, Translational Medicine Research Center, KUTTAM-NDAL Istanbul Turkey.
7. Division Translational Genomics of Neurodegenerative Diseases, Hertie-Institute for Clinical Brain Research and Center of Neurology, University of Tübingen, Tübingen, Germany.
8. Department of Pediatric Neurology, Centre for Neuromuscular Disorders, Centre for Translational Neuro- and Behavioral Sciences, University Duisburg-Essen, Essen, Germany.
9. Pediatric Neurology Research Group, Vall d'Hebron Research Institute, Universitat Autònoma de Barcelona, Barcelona, Spain.
10. Department of Clinical Neurosciences, University of Cambridge, Cambridge, UK.
11. Medical Research Council Mitochondrial Biology Unit, University of Cambridge, Cambridge, UK.
12. Institut du Cerveau, INSERM U1127, CNRS UMR7225, Sorbonne university, Paris, France
13. Center for Medical Genetics, Ghent University Hospital, Ghent, Belgium.
14. Program for Undiagnosed Rare Diseases (UD-PrOZA), Ghent University Hospital, Ghent, Belgium.
15. Department of Biomolecular Medicine, Faculty of Medicine and Health Sciences, Ghent University, Ghent, Belgium.
16. Neurology / Neurogenetics Laboratory University of Crete, Heraklion, Crete, Greece.

17. Institut National de la Santé et de la Recherche Medicale (INSERM) U1127, Paris, France.
18. Centre National de la Recherche Scientifique, Unité Mixte de Recherche (UMR) 7225, Paris, France.
19. Unité Mixte de Recherche en Santé 1127, Université Pierre et Marie Curie (Paris 06), Sorbonne Universités, Paris, France.
20. Centre de Référence de Neurogénétique, Hôpital de la Pitié-Salpêtrière, Assistance Publique-Hôpitaux de Paris (AP-HP), Paris, France.
21. Institut du Cerveau - ICM, Paris, France.
22. Department of Neurodegeneration, Hertie Institute for Clinical Brain Research (HIH), University of Tübingen, Tübingen, Germany.
23. German Center for Neurodegenerative Diseases (DZNE), Tübingen, Germany.
24. Institute of Medical Genetics and Applied Genomics, University of Tübingen, Tübingen, Germany.
25. Centre for Rare Diseases, University of Tübingen, Tübingen, Germany.
26. Dpt. of Neurology, Ghent University Hospital, Ghent, Belgium.
27. Institute of Systems Motor Science, University of Lübeck, Ratzeburger Allee 160, 23562, Lübeck, Germany.
28. Department of Neurology, University Hospital Schleswig Holstein, Ratzeburger Allee 160, 23562, Lübeck, Germany.
29. Department of Human Genetics, Radboud University Medical Center, Nijmegen, The Netherlands.
30. Department of Neurology and Epileptology, Hertie Institute for Clinical Brain Research (HIH), University of Tübingen, Tübingen, Germany.
31. Division of Neurodegenerative Diseases and Movement Disorders, Department of Neurology, University of Heidelberg, Heidelberg, Germany.
32. Institute of Neurogenetics, University of Lübeck, Ratzeburger Allee 160, 23562, Lübeck, Germany.
33. Institute of Neuroscience, Universitat Autònoma de Barcelona, Barcelona, Spain.
34. Department of Clinical and Movement Neurosciences, UCL Queen Square Institute of Neurology, University College London, WC1N 3BG.
35. Clinical Institute of Genomic Medicine, University Medical Centre Ljubljana, Slovenia.
36. Department of Human Genetics, Medical Research Institute, Alexandria University, Egypt.
37. U-703 Centre for Biomedical Research on Rare Diseases (CIBER-ER), Instituto de Salud Carlos III, Barcelona, Spain.
38. Movement Disorders Unit, Department of Child Neurology, Institut de Recerca Sant Joan de Déu.
39. European Reference Network for Rare Neurological Diseases (ERN-RND), Barcelona, Spain.
40. Department of Molecular Neurology, Friedrich-Alexander-Universität (FAU) Erlangen-Nürnberg, Erlangen, Germany.
41. Dementia Research Centre, Department of Neurodegenerative Disease, UCL Queen Square Institute of Neurology, London, UK.
42. Ecole Pratique des Hautes Etudes, Paris Sciences et Lettres Research University, Paris, France.
43. Peripheral Neuropathy Research Group, University of Antwerp, Antwerp, Belgium.
44. Department of Neurology, Radboud University Medical Center, Nijmegen, The Netherlands.
45. Donders Institute for Brain, Cognition and Behaviour, Radboud University Medical Center, Nijmegen, The Netherlands.
