# Supplementary material for: Advancing the diagnosis of rare neuromuscular and neurological diseases through the collaborative Solve-RD research framework
Source: J Neuromuscul Dis. 2026 Jul 15:22143602261460689. Online ahead of print. doi: 10.1177/22143602261460689 (PMC13438582; doi:10.1177/22143602261460689)
Supplement: Supplementary file 2 [file sj-pdf-2-jnd-10.1177_22143602261460689.pdf]

**Solve-RD DITF-EURO-NMD**

Özge Aksel-Kiliçarslan[1], Jonathan Baets[2-4], Rabah Ben Yaou[5, 6], Gisèle Bonne[5], Enrico Bugiardini[7], Ana Casasús[8], Patrick F Chinnery[9, 10], Kristl Claeys[11, 12], Enzo Cohen[5], Mireille Cossee[13, 14], Judith Cossins[15], Laura Costa[16], Jonathan de Winter[2-4], Jordi Diaz Manera[17], Berta Estevez[18, 19], Teresinha Evangelista[5], Alessandra Ferlini[20], Andrea Gangfuss[21], Fei Gao[9], David Gómez[16], Nora Gómez Lasarte[22], Peter Hackman[23], Rita Horvath[9], Henry Houlden[7], Mridul Johari[23], Danny Jomaa[24], Mert Karakaya[25-28], Jarred Lau[1], Hanns Lochmüller[1, 29, 30], Adolfo López de Munain Arregui[22], Will Macken[31], Chiara Marini-Bettolo[17], Pilar Marti[8], Manuela Morleo[32, 33], Juliane Müller[31], Francina Munell[16], Francesco Muntoni[31], Francesco Musacchia[32, 33], Andres Nascimiento Osorio[18], Daniel Natera[18], Isabelle Nelson[5], Vincenzo Nigro[32, 33], Catarina Olimpo[34], Aurelien Perrin[13, 14], Luke Perry[31], Giulio Piluso[32], Michele Pinelli[33, 35], Veronica Pini[31], Rob Pitceathly[31], Kiran Polavarapu[1], Alessia Pugliese[1], Penelope Romero[16], Andreas Roos[1, 21, 36], Amets Saenz Pena[22], Manuela Santos[37], Anna Sarkozy[31], Marco Savarese[23], Rita Selvatici[20], Malaichamy Sivasankar[1], Volker Straub[17], Rachel Thompson[1], Vincent Timmerman[3, 38], Ana Topf[17], Annalaura Torella[32, 33], Bjarne Udd[23, 39, 40], Liedewei van de Vondel[2, 3, 38], Charles van Goethem[13], Jana Vandrovcova[7], Alessandra Varavallo[33], Juan J Vilchez[8], Brunhilde Wirth[25-28], Irina Zaharieva[31], Miren Zulaica[22].

**Affiliations**

1. Children's Hospital of Eastern Ontario Research Institute, University of Ottawa, Ottawa, Canada.
2. Translational Neurosciences, Faculty of Medicine and Health Sciences, University of Antwerp, Belgium.
3. Laboratory of Neuromuscular Pathology, Institute Born-Bunge, University of Antwerp, Antwerpen, Belgium.
4. Neuromuscular Reference Centre, Department of Neurology, Antwerp University Hospital, Antwerpen, Belgium.
5. Sorbonne Université, Inserm, Institut de Myologie, Centre de Recherche en Myologie, F-75013 Paris, France.
6. Service des bases de données, Institut de Myologie, F-75013 Paris, France.
7. Department of Neuromuscular Diseases, UCL Queen Square Institute of Neurology, London, UK.
8. Health Research Institute Hospital La Fe, Valencia, Spain.
9. Department of Clinical Neurosciences, University of Cambridge, Cambridge, UK.
10. Medical Research Council Mitochondrial Biology Unit, University of Cambridge, Cambridge, UK.
11. Department of Neurology, University Hospitals Leuven, Leuven, Belgium.
12. Laboratory for Muscle Diseases and Neuropathies, Department of Neurosciences, and Leuven Brain Institute (LBI), KU Leuven - University of Leuven, Leuven, Belgium.
13. Laboratoire de Génétique Moléculaire, Centre Hospitalier Universitaire de Montpellier, 34093 Montpellier, France
14. PhyMedExp, Université de Montpellier, INSERM, CNRS, 34093 Montpellier, France.
15. Nuffield Department of Clinical Neurosciences, University of Oxford, UK.
16. Pediatric Neurology Research Group, Vall d'Hebron Research Institute, Universitat Autònoma de Barcelona, Barcelona, Spain.
17. John Walton Muscular Dystrophy Research Centre, Translational and Clinical Research Institute, Newcastle University and Newcastle Hospitals NHS Foundation Trust, Newcastle upon Tyne, UK.
18. Neuromuscular Disorders Unit , Department of Pediatric Neurology. Hospital Sant Joan de Déu, Barcelona, Spain

19. Laboratory of Neurogenetics and Molecular Medicine - IPER, Institut de Recerca Sant Joan de Déu, Barcelona, Spain.
20. Unit of Medical Genetics, Department of Medical Sciences, University of Ferrara, Italy.
21. Department of Pediatric Neurology, Developmental Neurology and Social Pediatrics, Children's Hospital University of Essen, Essen, Germany.
22. Hospital Universitario Donostia, San Sebastián, Spain.
23. Folkhälsan Research Centre and Medicum, University of Helsinki, Helsinki, Finland.
24. Queen’s School of Medicine, Queen’s University, Kingston, Ontario, Canada.
25. Institute of Human Genetics, University Hospital of Cologne, University Cologne, Kerpener Str. 34, 50931 Cologne, Germany.
26. Center for Molecular Medicine Cologne, University of Cologne, 50931 Cologne, Germany.
27. Institute for Genetics, University of Cologne, 50674 Cologne, Germany.
28. Center for Rare Diseases Cologne, University Hospital Cologne, 50937, Cologne, Germany.
29. Department of Neuropediatrics and Muscle Disorders, Medical Center, Faculty of Medicine, University of Freiburg, Freiburg, Germany.
30. Centro Nacional de Análisis Genómico (CNAG-CRG), Center for Genomic Regulation, Barcelona Institute of Science and Technology (BIST), Barcelona, Spain.
31. Dubowitz Neuromuscular Centre, UCL Great Ormond Street Hospital, London, UK.
32. Dipartimento di Medicina di Precisione, Università degli Studi della Campania "Luigi Vanvitelli", Napoli, Italy.
33. Telethon Institute of Genetics and Medicine, Pozzuoli, Italy.
34. Department of Clinical Genetics, Cambridge University Hospital, Cambridge, UK.
35. Università di Napoli Federico II, Napoli, Italy.
36. Department of Neurology, Heimer Institute for Muscle Research, University Hospital Bergmannsheil, Ruhr-University Bochum, 44789 Bochum, Germany.
37. Centro Hospitalar Universitário do Porto, Porto, Portugal.
38. Peripheral Neuropathy Research Group, University of Antwerp, Antwerp, Belgium.
39. Tampere Neuromuscular Center, Tampere, Finland.
40. Vasa Central Hospital, Vaasa, Finland.
